# Evaluation of the effectiveness of the model of family participatory care based on cloud follow-up in enhancing the quality of life and psychological well-being of patients with chronic difficult-to-heal traumatic tumours

**DOI:** 10.3389/fpubh.2026.1805462

**Published:** 2026-07-06

**Authors:** Dan Wang, Fengge Wang, Xin Wang, Yanling Chen, Saiqiong Zhong, Qiurong Pan

**Affiliations:** 1 Outpatient Department, The Fifth Affiliated Hospital, Southern Medical University, Guangzhou, Guangdong, China; 2 Emergency Medicine Department, The Fifth Affiliated Hospital, Southern Medical University, Guangzhou, Guangdong, China; 3 Guangzhou Conghua District Aotou Town Central Health Center, Guangzhou, Guangdong, China; 4 Nursing Department, The Fifth Affiliated Hospital, Southern Medical University, Guangzhou, Guangdong, China

**Keywords:** chronic difficult-to-heal tumours, cloud follow-up, family participatory care, mental health, quality of life, tumours

## Abstract

**Background:**

Patients with chronic difficult-to-heal traumatic tumours are prone to comorbid anxiety and depression. The clinical application of the cloud-based family engagement care model for these tumours remains under intensive investigation.

**Objective:**

This study sought to explore the impacts of the cloud-based family engagement care model on quality of life and mental health outcomes among patients with chronic difficult-to-heal traumatic tumours.

**Methods:**

This is a retrospective observational controlled study. Data were collected from 60 patients diagnosed with chronic difficult-to-heal traumatic tumours who were admitted to our institution between January 2023 and December 2024. Participants were stratified into the usual care group (receiving standard clinical care) and the cloud-based family engagement care group (undergoing cloud-facilitated follow-up integrated with family-participated care). After propensity score matching (PSM), 25 patients were included in each group. Primary outcome measures encompassed total scores of the Hospital Anxiety and Depression Scale (HADS) and pain intensity evaluated via the Visual Analog Scale (VAS). Secondary outcomes included the 36-Item Short Form Health Survey (SF-36) scores, wound healing duration, wound contraction rate, exudate severity, odor intensity, and sleep quality.

**Results:**

Following PSM, the baseline characteristics of the two groups were balanced and comparable (all P>0.05). Patients in the cloud-based family engagement care group demonstrated substantially better performance in quality of life-related indicators, and wound contraction rate compared with those in the usual care group (all P<0.05). Additionally, the cloud-based family engagement care group exhibited notably lower HADS anxiety scores, HADS depression scores, VAS pain scores, shorter wound healing time, milder wound exudate, less prominent wound odor, and improved sleep quality relative to the usual care group (all P<0.05).

**Conclusion:**

Compared with usual care, the cloud-based family engagement care model significantly improves quality of life and mental health in these patients, showing great potential for clinical application and promotion.

## 1 Introduction

Patients with chronic, difficult-to-heal traumatic tumours are a special group of patients who face significant clinical challenges. Their traumas often remain open due to tumour progression, side effects of radiotherapy or immune suppression, resulting in long-term pain, infection and dysfunction (1). Despite recent advances in diagnostic and therapeutic approaches, this group of patients not only suffers from severely impaired physical functioning, but also from poor mental health, as studies have shown that more than 60% of patients suffer from moderate-to-severe anxiety or depression, which significantly reduces treatment adherence and quality of life (2). The traditional medical follow-up model is limited by the temporal and spatial limitations of outpatient follow-up, which makes it difficult to provide patients with continuous and dynamic monitoring of their conditions and personalized care guidance, especially during the critical recovery period after discharge from hospitals, which often results in a breakdown of care support and a lack of self-management ability (3). With the in-depth promotion of the strategy of “Internet+Healthcare,” the cloud follow-up platform based on health technology provides a new solution to break through this dilemma, which realizes the whole process of chronic trauma management through remote vital signs monitoring, trauma image transmission, and online doctor-patient communication etc (4). However, the current research on the application of cloud follow-up in tumour-associated difficult-to-heal tumours is still in its infancy, and its actual effect and mechanism of action need to be evaluated.

Family participatory care (FCC), as a new model of care centered on the patient and family, has demonstrated unique advantages in chronic disease management (5). By systematically integrating family members into the care team, this model not only provides continuous emotional support, but also improves family caregiving ability through professional nursing skills training, thus enhancing patients’ self-management efficacy and confidence in recovery (6). It is worth noting that in the long-term management of patients with traumatic tumours, the quality of the family support system has a direct impact on the patient’s psychological adjustment and wound healing, but the integration of family care resources is still insufficient in clinical practice (7). The combination of cloud follow-up technology and FCC model through a digital platform that enables professional medical teams and family care is expected to build a collaborative management network of. The combination of cloud follow-up technology and FCC model through a digital platform that enables professional medical teams and family care is expected to build a collaborative management network of “hospital-family-patient” (8). This innovative model can improve the comprehensive prognosis of patients with chronic difficult-to-heal traumatic tumours by leveraging the technological advantages of cloud follow-up in remote monitoring and data sharing, and by activating the emotional and functional values of the family support system (9). The aim of this study was to systematically analyze the impact of this integrated intervention the impact on the quality of life of patients, psychological well-being and wound healing, with a view to providing a high-level evidence-based basis for optimizing the continuity of care for chronic traumatic tumours, as well as a theoretical reference for the innovation of personalized care models in the digital healthcare era.

## 2 Materials and methods

### 2.1 Study design and participants

**Figure 1:**
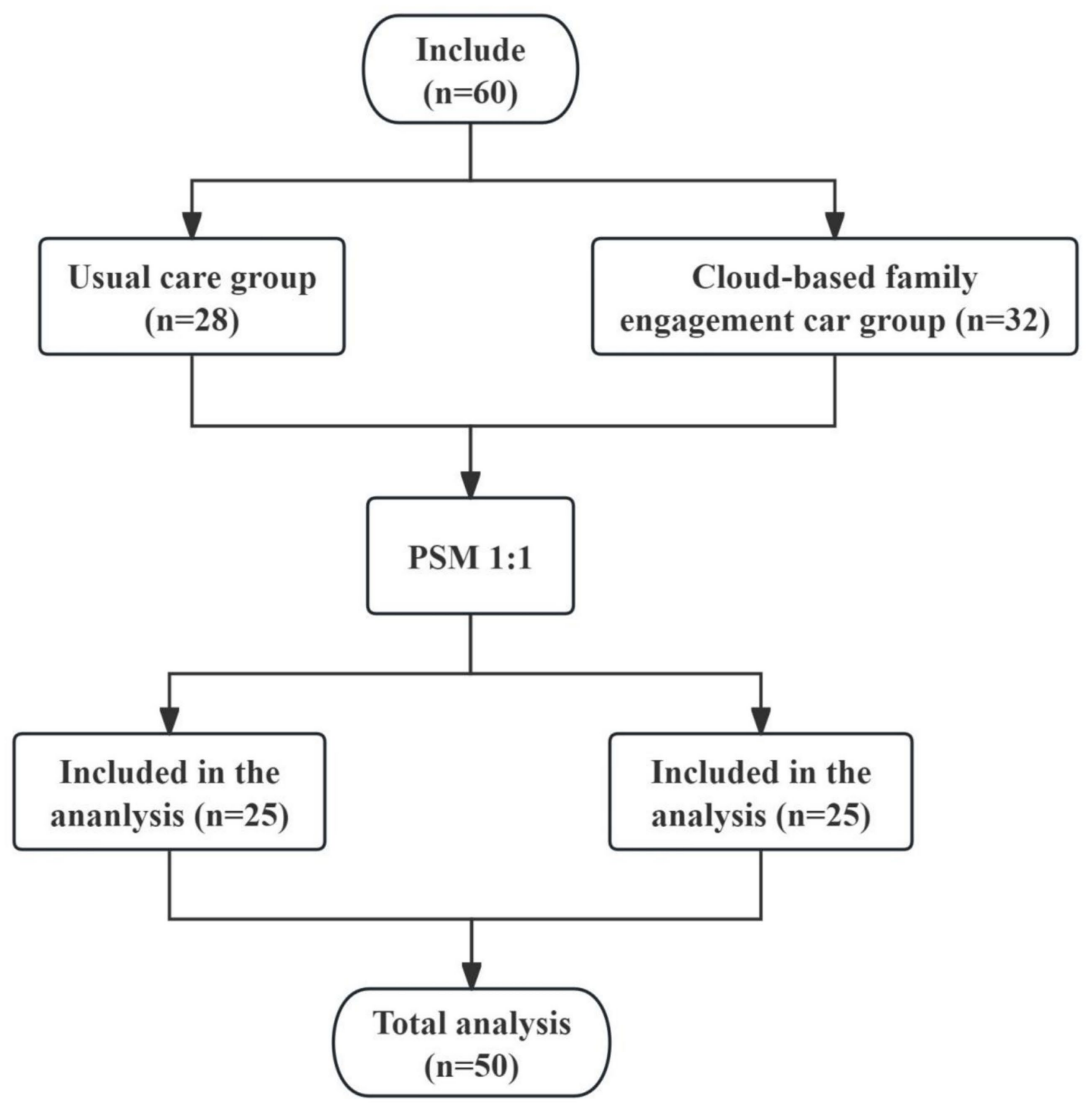
Flowchart of the study.

This is a retrospective observational controlled study designed to evaluate the efficacy of the cloud-based family engagement care model in improving quality of life and mental health among patients with chronic difficult-to-heal traumatic tumours. Medical records of patients diagnosed with chronic difficult-to-heal traumatic tumours admitted to the Oncology Center of a Grade A tertiary hospital between January 2023 and December 2024 were retrospectively analyzed. A total of 60 patients meeting the initial inclusion criteria were screened via the hospital’s electronic medical record (EMR) system, and 10 patients were excluded based on the exclusion criteria. Ultimately, 50 patients were enrolled and divided into two groups according to the care modality: the cloud-based family engagement care group (*n* = 25, receiving cloud-based follow-up integrated with family-participated care) and the usual care group (*n* = 25, receiving conventional follow-up care; Figure 1).

### 2.2 Inclusion, and exclusion criteria

Inclusion Criteria: (1) Patients with a pathologically confirmed malignant tumor; (2) Presence of tumor-associated chronic refractory traumatic tumours with a wound duration of ≥6 weeks and no tendency for spontaneous epithelialization; (3) Age ranging from 18 to 80 years; (4) Documented continuous treatment duration of no less than 6 months in this hospital; (5) Complete and traceable medical records, including wound assessment, scale evaluation, and follow-up data (10).

Exclusion Criteria: (1) Comorbidity with severe cognitive impairment; (2) Death during the treatment period; (3) Missing data for key outcome measures (quality of life scales, mental health scales, wound assessment records) exceeding 30% (11).

### 2.3 Ethical statement

This study was conducted in strict adherence to the Declaration of Helsinki and the relevant guidelines of The Fifth Affiliated Hospital, Southern Medical University Institutional Review Board, and has been approved by the IRB (Approval No.: IT 【2022】-A040).

### 2.4 Study methodology

A retrospective observational controlled study design was adopted, utilizing EMR data of patients with chronic difficult-to-heal traumatic tumours admitted to our hospital. Patients were stratified into two groups based on the care modality documented in their medical records: the cloud-based family engagement care group (*n* = 25) received a novel intervention (cloud-based follow-up combined with family-participated care), while the usual care group (*n* = 25) received conventional follow-up care. Baseline data (e.g., age, gender, body mass index) were collected to investigate the effects of the cloud-based family engagement care model on patient-reported outcomes (PROs) and mental health status. Outcome measures included quality of life, psychological status, wound healing promotion, reduction in complications, Visual Analog Scale (VAS) pain scores, and nursing satisfaction.

### 2.5 Intervention protocols

Cloud-based Family Engagement Care Group (Cloud-based Follow-up + Family-participated Care).

#### 2.5.1 Cloud follow-up platform

(1) Wound monitoring: Specialized nurses provided one-on-one offline training within 24 h after discharge to standardize wound photography (15–20 cm vertical shot, natural indoor light without flash, full coverage of wound margin, exudate and surrounding skin). Patients and relatives uploaded weekly high-definition wound photos with assessment records; nurses reviewed images within 2 working hours, marked abnormal signs and archived serial wound data in real time.
(2) Symptom and medication recording: Participants filled in daily morning and evening VAS scores (0–10) and medication information via visualized platform templates. Nurses checked data at 18:00 daily and sent reminders for incomplete records.
(3) Online consultation: Patients could submit questions anytime; a multidisciplinary team (attending physicians, wound nurses, pharmacists) offered centralized replies every Tuesday and Friday afternoon, while a 24-h emergency channel was available for severe complications, with all consultations electronically documented.
(4) Intelligent reminders: Three-mode reminders (platform popup, SMS, WeChat mini-program) were delivered 24 h before dressing change, at fixed medication time daily, and 3 working days prior to outpatient visits. Nurses followed up unread alerts by phone, achieving a 100% reminder response rate (12).

#### 2.5.2 Family-participated care

(1) Standardized training: A 90-min offline training within 48 h of admission covered wound care skills and psychological support with simulated wound models; online teaching videos were accessible on the cloud. All family members passed written (≥80 points) and practical examinations; unqualified participants received repeated one-on-one tutoring.
(2) Weekly family care meetings: Hosted by attending physicians with nurses, therapists and immediate family members (20–30 min per session), meetings adjusted personalized care plans according to weekly wound recovery, pain and lifestyle data, clarified family care responsibilities, and kept dual electronic and paper medical archives.
(3) Assisted data entry: Caregivers completed platform-based pain/medication logs and wound photo uploading, serving as primary operators for older adults or immobile patients. Nurses conducted weekly random inspections to correct irregular operations timely.

#### 2.5.3 Usual care group (conventional follow-up)

(1) Outpatient visits every 4 weeks: Doctors performed physical wound assessment, VAS measurement and medication adjustment, with all data manually recorded on paper charts without electronic backup.
(2) Biweekly telephone follow-up (14:00–17:00 on workdays, 5–10 min per call): Nurses collected subjective symptoms by questionnaires without wound photographs, and filed follow-up results in nursing registers.
(3) General health education was delivered via verbal guidance and uniform brochures without customized training for family members, who provided voluntary daily assistance without standardized wound nursing training or regular behavioral supervision (13).

### 2.6 Data extraction

Data were retrospectively retrieved from electronic medical records, cloud follow-up platform, follow-up files and specialized management documents following standard retrospective research protocols.

Before data collection, researchers developed a standardized extraction checklist specifying variables, formats, units and missing-data rules. Subjects were initially screened per inclusion/exclusion criteria to exclude patients with incomplete files, lost to follow-up or severe multi-organ dysfunction. Two trained oncology nurses and one statistician performed blinded independent data extraction unaware of group assignment to minimize selection and information bias.

All raw data were double-entered into EpiData 3.1 for consistency verification. Built-in logical checks plus manual cross-review screened duplicates, outliers and logical errors; questionable values were verified against original medical charts, corrected or excluded with documented reasons. Source materials were preserved electronically and on paper for full traceability.

Baseline data (demographics, clinical features and initial assessments) were abstracted from admission charts within 1 week before treatment; baseline was defined as platform enrollment day for the intervention group and first outpatient visit for controls. Short-term endpoints at 6 months covered wound exudation, pain and sleep status. A statistician coded and categorized all outcomes by variable type for subsequent analyses. The full dataset was saved in an encrypted password-limited database accessible exclusively to the research team to guarantee confidentiality and data integrity.

### 2.7 Outcome measures

#### 2.7.1 Primary outcome measures

(1) Hospital Anxiety and Depression Scale (HADS): Used to assess psychological distress, with each item scored on a 0–3 scale and a total score ranging from 0 to 21. Higher scores indicate more severe anxiety and depression symptoms.
(2) Visual Analog Scale (VAS) Pain Score: Assesses pain intensity on a 0–10 scale, with higher scores indicating more severe pain (14).

#### 2.7.2 Secondary outcome measures

(1) 36-Item Short Form Health Survey (SF-36): A multidimensional health status assessment tool covering 8 domains: Physical Functioning, Role-Physical, Bodily Pain, General Health, Vitality, Social Functioning, Role-Emotional, and Mental Health. Each domain is scored as a percentage, with higher scores indicating better health status.
(2) Wound healing time: Defined as the total number of days from intervention initiation to complete wound epithelialization (no exudate, eschar shedding), recorded during regular wound inspections (daily or every other day) by medical staff.
(3) Wound contraction rate: For regular-shaped tumours, area was calculated using length and width measurements with a ruler; for irregular-shaped tumours, area was determined via transparent grid tracing or digital photography combined with image analysis software. The wound contraction rate was calculated as follows: (1 - Current wound area / Initial wound area) × 100%. The incidence of wound infection was also recorded.
(4) Wound exudate severity: Assessed by medical staff based on dressing saturation and scored using a standardized grading scale.
(5) Wound odor intensity: Rated by patients or medical staff on a 0–10 scale (0 = no odor, 10 = intolerable odor), with higher scores indicating a stronger dose-dependent impairment of quality of life.

Standardized assessment procedure for subjective wound indicators: To reduce measurement bias of subjective wound evaluation, all assessments of wound exudate and odor were performed following unified international wound care guidelines. Exudate severity was classified by dressing saturation (mild: <25%; moderate: 25–75%; severe: >75%). All evaluations were conducted under consistent environmental conditions with fixed temperature, ventilation and evaluation time. Two professionally trained wound care nurses independently completed all wound assessments. Discrepancies were resolved by joint re-evaluation. The Cohen’s Kappa values for exudate and odor assessment were 0.812 and 0.845, respectively, indicating excellent inter-rater reliability. All evaluators received unified training for scoring criteria before data collection to ensure evaluation consistency.

(1) Sleep quality: Evaluated using the Pittsburgh Sleep Quality Index (PSQI) via a standardized self-reported questionnaire, with higher scores indicating poorer sleep quality.
(2) Self-reported appetite score: Documented as a secondary subjective outcome measure.

### 2.8 Statistical analysis

Statistical analyses were performed using SPSS 27.0 software. All demographic and clinical baseline indicators listed in Table 1 were included as covariates for propensity score matching (PSM) in this study. The specific matching variables consisted of age, gender, body mass index (BMI), educational level, TNM tumor stage, wound trauma area, radiotherapy history, chemotherapy history, analgesic use, SF-36 physical component score, HADS anxiety score, and VAS pain score. Propensity score matching (PSM) was conducted using nearest neighbor matching with a caliper of 0.02. The standardized mean difference (SMD) was used to assess baseline balance between groups, with SMD < 0.1 indicating good balance. Normality of data was tested using the Shapiro–Wilk test. Normally distributed continuous data were presented as mean ± standard deviation (mean ± SD), with paired t-tests for intragroup comparisons and independent samples t-tests for intergroup comparisons. Non-normally distributed continuous data were presented as median (interquartile range) [M (Q1, Q3)], with Wilcoxon signed-rank tests for intragroup comparisons and Mann–Whitney U tests for intergroup comparisons. Categorical data were presented as n (%), with Chi-square tests for intergroup comparisons. All statistical tests were two-tailed, and a *p*-value < 0.05 was considered statistically significant. The *p*-values were adjusted using the Bonferroni correction.

**Table 1:** Comparison of patients’ general information.

sports event	Cloud-based family engagement care group (*n* = 25)	Usual care group (*n* = 25)	*χ^2^/t*	*p*-value	Shapiro–Wilk	Effect size	SMD
Age (years, $\bar{x}$ ±s)	58.3 ± 12.1	57.6 ± 11.8	0.208	0.836	0.803/0.799	Cohen’s D = 0.059	0.058
Sex (m/f, example)	14/11	13/12	0.081	0.777	–	Phi = 0.040	−0.114
BMI (kg/m^2^, $\bar{x}$ ±s)	22.2 ± 3.1	22.6 ± 2.5	−0.516	0.608	0.641/0.939	Cohen’s D = -0.146	−0.142
Educational level (high school and above, %)	60.0(15/25)	56.0 (14/25)	0.082	0.774	–	Phi = 0.041	−0.115
TNM (stage III/IV, %)	72.0 (18/25)	68.0 (17/25)	0.095	0.758	–	Phi = 0.044	−0.123
Trauma area (cm^2^, $\bar{x}$ ±s)	15.8 ± 8.8	16.1 ± 6.9	−0.130	0.897	0.565/0.995	Cohen’s D = -0.037	−0.038
Received radiotherapy (%)	76.0 (19/25)	72 (18/25)	0.104	0.747	–	Phi = 0.046	−0.129
Received chemotherapy (%)	84.0 (21/25)	80.0 (20/25)	0.136	0.713	–	Phi = 0.052	−0.147
Use of analgesics (%)	64.0 (16/25)	60.0 (15/25)	0.085	0.771	–	Phi = 0.041	−0.117
SF-36 Physiological Health (points)	42.1 ± 8.7	43.0 ± 7.8	−0.377	0.708	0.210/0.959	Cohen’s D = -0.107	−0.109
HADS Anxiety (points)	8.8 ± 3.2	8.8 ± 2.9	<0.001	1.000	0.146/0.836	Cohen’s D < 0.001	<0.001
VAS pain (points)	5.7 ± 1.3	5.6 ± 1.6	0.393	0.696	0.214/0.205	Cohen’s D = 0.111	0.069

BMI, Body mass index; TNM, Tumour stage; SF-36, 36-item Short Form Health Survey; HADS, Hospital Anxiety and Depression Scale; VAS, Visual analog scale; SMD, Standardized Mean Difference.

## 3 Results

### 3.1 Comparison of general information

A total of 60 patients were initially enrolled in this study, and after propensity score matching (PSM), 50 patients were included in the final analysis with 25 cases allocated to each group. All standardized mean differences (SMD) were less than 0.2, indicating satisfactory baseline balance between the two groups. Statistical comparisons revealed no statistically significant differences in demographic characteristics or baseline data between the two groups (all *p* > 0.05), indicating that the patients were comparable at the initiation of treatment, with detailed data presented in Table 1.

### 3.2 Comparison of quality of life indicators

Comparative data on quality of life indicators are detailed in Table 2. Study findings demonstrated that all metrics in the cloud-based family engagement care group were significantly enhanced compared with those in the usual care group, with statistical significance (*p* < 0.05). Specifically, the cloud-based family engagement care group achieved substantially higher scores across all SF-36 domains relative to the usual care group (*p* < 0.05). These results indicate that the cloud-based follow-up integrated with family engagement care model effectively ameliorates patients’ activities of daily living and work functioning.

**Table 2:** Quality of Life Indicators Comparison ( $\bar{x}$ *± s*, points).

Dimension (math.)	Cloud-based family engagement care group (*n* = 25)	Usual care group (*n* = 25)	*t*	*p*	Shapiro–Wilk	Effect size (Cohen’s D)
Physiological function	72.4 ± 10.6	58.3 ± 12.8	4.250	<0.001	0.410/0.398	1.202
Physical function	68.5 ± 11.3	55.8 ± 13.5	3.617	<0.001	0.369/0.375	1.023
Pain in the body	75.3 ± 9.8	62.4 ± 11.3	4.310	<0.001	0.367/0.378	1.219
General health	65.2 ± 8.7	56.2 ± 10.4	3.328	0.002	0.329/0.366	0.941
Energies	70.6 ± 7.8	60.8 ± 9.5	3.969	<0.001	0.273/0.355	1.123
Social function	74.8 ± 8.3	63.5 ± 10.1	4.318	<0.001	0.368/0.409	1.221
Emotional function	71.5 ± 9.3	59.7 ± 11.6	3.968	<0.001	0.404/0.406	1.122
Mental health	73.7 ± 7.7	62.3 ± 9.8	4.566	<0.001	0.341/0.367	1.291

### 3.3 Comparison of mental state indicators

Comparative findings of psychological status metrics are presented in Table 3. The cloud-based family engagement care group exhibited significantly lower Hospital Anxiety and Depression Scale (HADS) anxiety and depression subscale scores compared to the usual care group (*p* < 0.001), indicating that the model effectively alleviated patients’ feelings of tension and anxiety through telepsychological counseling and family companionship.

**Table 3:** Comparison of mental status indicators in patients ( $\bar{x}$ *±s*, points).

norm	Cloud-based family engagement care group (*n* = 25)	Usual care group (*n* = 25)	*t*	*p*	Shapiro–Wilk	Effect size (Cohen’s D)
HADS Anxiety	4.8 ± 2.3	7.6 ± 2.9	−3.731	<0.001	0.340/0.150	−1.055
HADS depression	4.4 ± 1.8	7.0 ± 2.5	−4.266	<0.001	0.100/0.239	−1.207

### 3.4 Comparison of indicators for promoting wound repair and reducing complications

Comparative outcomes of wound healing metrics are detailed in Table 4. The cloud-based family engagement care group had a wound healing time of 32.4 ± 7.8 days, which was significantly shorter than that of the usual care group (45.5 ± 9.3 days, *p* < 0.001). This finding suggests that remote wound monitoring combined with standardized family care exerts a synergistic effect in accelerating tissue repair processes.

**Table 4:** Comparison of indicators for promoting wound repair and reducing complications.

Norm	Cloud-based family engagement care group (n = 25)	Usual care group (*n* = 25)	*χ^2^/t*	*p*	Shapiro–Wilk	Effect size (Cohen’s D)
Healing time (days)	32.4 ± 7.8	45.5 ± 9.3	−5.400	<0.001	0.749/0.576	−1.527
Wound reduction rate (%)	82.6 ± 10.5	61.3 ± 14.2	6.028	<0.001	0.512/0.660	1.705

Furthermore, the cloud-based family engagement care group attained a significantly higher wound contraction rate of 82.6 ± 10.5% compared to the usual care group (61.3 ± 14.2%, *p* < 0.001). This result demonstrates that family care guided by the cloud-based platform can more efficiently promote the progression of wound epithelialization.

### 3.5 Comparison of VAS scores and satisfaction with care

Comparative results of Visual Analog Scale (VAS) pain scores are presented in Table 5. The cloud-based family engagement care group exhibited a significantly lower VAS pain score of 3.0 ± 1.5 points compared to the usual care group (5.3 ± 1.2 points, *p* < 0.001). This outcome indicates that the intervention protocol integrating a remote pain monitoring system with family-assisted medication administration yields a marked therapeutic effect, reducing patients’ pain intensity from “moderate pain” to “mild pain”.

**Table 5:** Comparison of VAS scores and satisfaction with care.

norm	Cloud-based family engagement care group (*n* = 25)	Usual care group (*n* = 25)	*χ^2^/t*	*p*	Shapiro–Wilk	Effect size (Cohen’s D)
VAS Pain Score (points)	3.0 ± 1.5	5.3 ± 1.2	−6.148	<0.001	0.484/0.096	−1.739

### 3.6 Comparison of indicators of improvement of invasive condition and relief of systemic symptoms

Comparative data on wound condition improvement and sleep quality are detailed in Table 6. Regarding wound-related outcomes: The cloud-based family engagement care group exhibited a significantly lower wound exudate severity score of 1.0 (1.0,2.0) points compared to the usual care group [2.0 (1.5,2.0) points, *p* = 0.002], suggesting that remote wound management guidance effectively controls inflammatory exudation. Additionally, the wound odor score in the cloud-based family engagement care group was 2.1 ± 1.1 points, which was significantly lower than that in the usual care group (4.4 ± 1.3 points; *p* < 0.001). This reflects that the intervention enhances wound cleanliness and improves infection control efficacy.

**Table 6:** Comparison of indicators of improvement in invasive condition and relief of systemic symptoms ( $\bar{x}$ *± s*).

norm	Cloud-based family engagement care group (*n* = 25)	Usual care group (*n* = 25)	*Z/t*	*p*	Shapiro–Wilk	Effect size (Cohen’s D)
Degree of wound exudation (0–3 points)	1.0 (1.0,2.0)	2.0 (1.5,2.0)	−3.127	0.002	0.003/<0.001	-
Wound odor (VAS score 0–10)	2.1 ± 1.1	4.4 ± 1.3	−6.800	<0.001	0.051/0.080	−1.923
Sleep Quality (PSQI)	6.5 ± 2.7	9.5 ± 2.7	−3.884	<0.001	0.115/0.653	−1.099

PSQI, Pittsburgh Sleep Quality Index.

With regard to sleep quality, the cloud-based family engagement care group achieved a significantly better score of 6.5 ± 2.7 points compared to the usual care group (9.5 ± 2.7 points, p < 0.001), indicating that pain relief and emotional improvement exert a positive impact on sleep quality.

## 4 Discussion

Patients with chronic difficult-to-heal traumatic tumours face unique clinical challenges. Due to factors such as the inherent biological characteristics of tumors, radiation therapy side effects, and immunosuppression, their wound healing process is often significantly delayed (5). These patients not only endure physical distress including persistent pain and recurrent infections but also frequently develop psychological disorders such as anxiety and depression, which severely compromise their quality of life (15). Under the traditional medical model, post-discharge wound management primarily relies on regular outpatient follow-up. This fragmented care approach fails to achieve continuous wound monitoring and timely intervention (16). In recent years, with the advancement of digital health technology, cloud-based follow-up platforms have emerged as a novel solution for chronic disease management. By integrating remote monitoring, data sharing, and online consultation functions, these platforms effectively overcome the temporal and spatial limitations of traditional healthcare (17). However, technology-only remote interventions often overlook the critical role of family support systems in chronic disease management. Family-participated care emphasizes integrating family members into the care team to improve care quality through emotional support and skill training, yet its application in tumor-associated wound management remains insufficiently explored (18). Combining the technical advantages of cloud-based follow-up with the family-participated care model to construct a “hospital-family-patient” collaborative management framework may provide an innovative approach to improving the comprehensive prognosis of patients with chronic difficult-to-heal traumatic tumours. Based on this premise, the present study aimed to evaluate the efficacy of the cloud-based family engagement care model in enhancing quality of life and mental health among these patients, thereby providing evidence-based support for optimizing clinical decision-making.

Patients with chronic difficult-to-heal traumatic tumours often experience severe physical dysfunction and psychosocial adaptation issues due to prolonged wound non-healing, recurrent infections, and extended treatment cycles (19). A study by Huang et al. demonstrated that family nursing training reduced the incidence of wound infections by 18.7% (20). Consistent with this finding, the results of the present study showed that patients in the cloud-based family engagement care group exhibited significantly superior performance in physical function, mental health, and social function compared to the usual care group (all *p* < 0.05), confirming the effectiveness of this innovative care model. Notably, although the current study yielded relatively positive intervention effects with relatively large effect sizes, these clinical improvements are clinically plausible and can be reasonably explained by the multidimensional advantages of the optimized care model. Different from conventional single-point outpatient or telephone follow-up, the cloud-family collaborative model realizes full-cycle, uninterrupted wound monitoring, standardized home wound care, real-time symptom intervention, and sustained emotional support, which targets the core clinical pain points of patients with refractory traumatic tumour wounds, including delayed healing, uncontrolled symptoms, and insufficient psychological support. Such systematic and holistic intervention fundamentally makes up for the deficiencies of fragmented traditional care, thereby producing substantial clinical benefits. Nevertheless, considering the inherent limitations of single-center retrospective design and limited PSM-matched sample size, these favorable outcomes should be interpreted prudently rather than overgeneralized. The prominent intergroup differences observed in this study may not be solely attributed to the intervention itself, and potential residual confounding factors cannot be completely excluded. The intervention group demonstrated notable advantages in both physiological domains (e.g., physical function, bodily pain) and psychosocial domains (e.g., social function, mental health). This comprehensive improvement indicates that the cloud-based family engagement care model can enhance patients’ overall health status across multiple dimensions. Furthermore, the significant improvements in bodily pain and mental health observed in this study suggest potential underlying mechanisms: timely pain assessment and intervention by family members, remote medication guidance from healthcare professionals, and psychological interventions to modulate patients’ pain perception. Additionally, continuous psychological support provided via cloud-based follow-up, an enhanced emotional support system through family participation, and positive psychological feedback from symptom improvement may collectively contribute to the amelioration of patients’ physical and mental well-being. Collectively, these findings indicate that the cloud-based family engagement care model can significantly improve the quality of life and mental health of patients with chronic difficult-to-heal traumatic tumours, yielding comprehensive benefits in physical, psychological, and social functioning.

As medical models evolve, the cloud-based family engagement care model has provided a novel intervention pathway for improving patients’ mental health by integrating telemedicine technology with family support systems (21). Patients with chronic difficult-to-heal traumatic tumours commonly experience significant anxiety, depression, and psychological trauma (22), yet the traditional biomedical model often overlooks their mental health needs, resulting in low levels of post-traumatic growth (23). A path analysis study by Yan et al. on hospitalized patients with chronic trauma revealed that social support could indirectly improve quality of life by regulating anxiety levels (*β* = −0.32) (24). The results of the present study showed that patients in the cloud-based family engagement care group had significantly reduced anxiety and depression scores (*p* < 0.001), which may be closely associated with continuous psychological support via cloud-based follow-up, emotional companionship and encouragement from family members, and positive psychological feedback from symptom improvement. Furthermore, the significant increase in total Post-Traumatic Growth Inventory (PTGI) scores among patients in the intervention group indicates that the intervention not only alleviates negative emotions but also fosters positive psychological changes. The obvious psychological benefits observed in this study are clinically reasonable. Patients with long-term refractory tumour wounds suffer from persistent physical discomfort and disease uncertainty, which easily induce negative emotions. The continuous online psychological guidance and stable family emotional companionship provided by the intervention model effectively fill the gap of long-term psychological management in traditional care. However, due to the non-randomized retrospective nature of this study and limited sample capacity, the psychological improvement effects with large effect sizes need cautious interpretation. It cannot be ruled out that unmeasured individual differences in psychological resilience and family care atmosphere partially amplify the intervention effects. Therefore, the cloud-based family engagement care model can effectively improve anxiety and depression symptoms and promote post-traumatic growth in patients with chronic difficult-to-heal traumatic tumours.

With the advancement of telemedicine technology, the cloud-based family engagement care model has offered a novel solution for improving wound healing outcomes by integrating professional medical guidance with family care support (25). Leveraging real-time wound monitoring, remote professional guidance, and standardized family care, this model is expected to significantly enhance the quality and efficiency of wound management (26). The present study compared the wound healing outcomes between the cloud-based family engagement care model and the traditional care model. The results showed that patients in the cloud-based family engagement care group had significantly shorter wound healing time, higher wound contraction rate (both *p* < 0.001), and a notably lower infection rate. From a clinical perspective, the significant improvement in wound healing indicators is highly consistent with the practical logic of wound management. Refractory traumatic tumour wounds require long-term, standardized, and dynamic nursing management. The traditional intermittent follow-up mode cannot timely identify wound deterioration risks such as increased exudation and local infection, while the cloud-based real-time monitoring combined with standardized family home care achieves early warning and early intervention of wound problems, which is the core reason for the optimized wound healing outcomes. This fully verifies the clinical rationality of the intervention effect. Nonetheless, considering the single-center setting and limited paired samples after PSM, the robust wound-improving effects observed in this study should be recognized as preliminary findings. The efficacy and popularization value of the model still need further verification through larger-sample and multicenter studies to avoid overinterpretation of the current results. These findings indicate that real-time wound monitoring via cloud-based follow-up, timely intervention and guidance from healthcare professionals, and standardized daily care provided by family members offer significant advantages in preventing wound infections, improving treatment adherence, optimizing wound care quality, and enhancing patients’ self-management capabilities. Thus, this model can significantly improve wound healing outcomes, shorten healing time, and increase wound contraction rate in patients with chronic difficult-to-heal traumatic tumours.

Patients with chronic difficult-to-heal traumatic tumours often experience severe impairment of quality of life due to clinical symptoms such as wound exudate and odor, which can also trigger systemic issues including sleep disorders and decreased appetite (27). The traditional wound care model focuses on local wound management and often overlooks the impact of these symptoms on patients’ overall health status (28). With the popularization of holistic nursing concepts, the cloud-based family engagement care model has opened a new pathway for improving wound-related symptoms and enhancing patients’ overall quality of life by integrating remote monitoring, symptom management, and family support (29). A study by Chen et al. demonstrated that a cloud-based platform-guided exudate management protocol reduced wound exudate volume by 42% and decreased the incidence of wound odor from a baseline of 68 to 29% (30). The present study systematically compared the differences in wound symptom control and systemic health improvement between the cloud-based family engagement care group and the usual care group. It was found that patients in the intervention group had significantly reduced wound exudate severity and lower odor scores, which may be closely related to early exudate intervention enabled by remote monitoring and standardized wound care performed by family members. Meanwhile, patients in the cloud-based family engagement care group exhibited significantly improved sleep quality (*p* = 0.001), indicating that the model not only improves local wound symptoms but also promotes a positive cycle of overall health status. The systematic improvement in local wound symptoms and systemic sleep status further confirms the clinical rationality of the intervention. Local persistent wound symptoms are the direct cause of poor sleep and reduced quality of life in such patients. The model realizes precise and continuous management of wound exudate, odor and other symptoms, thereby fundamentally alleviating physical discomfort and indirectly improving systemic health status. However, in view of the methodological limitations of this retrospective study, the superior comprehensive improvement effect should be interpreted cautiously. The observed large effect sizes may be affected by residual confounding factors, and the conclusion is only applicable to the enrolled population of this single-center study, without widespread generalization for the time being. Consequently, the cloud-based family engagement care model can significantly alleviate wound exudate, odor, and other symptoms, effectively enhance sleep quality and appetite, achieving a transition from local wound management to comprehensive improvement of patients’ systemic health.

## 5 Study limitations

This study has several unavoidable limitations. First and foremost, there are inherent limitations of the study design. As a retrospective observational controlled study, although PSM was applied to balance baseline characteristics, this study was limited by unmeasured factors such as digital literacy, family support, and treatment adherence, compared to randomized controlled trials (RCTs), it cannot fully eliminate selection bias and information bias. In particular, patients in this study were grouped based on the actual care modality they received rather than random assignment, which may have led to differences in unmeasured characteristics between the two groups. Second, attention bias may have influenced the results. Patients in the cloud-based family engagement care group uploaded wound photographs weekly, recorded daily pain scores, received family training, and participated in weekly family care conferences, whereas those in the usual care group only underwent outpatient follow-up every 4 weeks and telephone follow-up every 2 weeks. This difference in interaction frequency itself may have exerted a positive impact on patients, including increased attention, enhanced treatment confidence, and improved adherence. These factors may have influenced the study results independently of the intervention itself. Thirdly, there were difficulties in implementing blinding. Due to the specific nature of the intervention, blinding of patients, family members, and healthcare providers was not feasible. Patients and their families were fully aware of their group allocation, and healthcare providers were also informed of the grouping during intervention implementation. Although this open-label design facilitated intervention delivery, it increased the risk of expectation bias. Particularly in the assessment of subjective outcomes such as pain scores and nursing satisfaction, the expectations of patients and assessors may have compromised the objectivity of the results. Fourthly, the study was limited by a relatively small sample size. A total of 50 patients were ultimately enrolled, which may have resulted in insufficient statistical power. This could lead to failure to detect some true effects or, conversely, false identification of accidental differences as statistically significant. Fifthly, the follow-up period was limited. The study required patients to complete at least 6 months of follow-up, but this time frame may not be sufficient to evaluate certain long-term effects. The healing process of chronic refractory tumours is often prolonged, and improvements in mental health may require a longer period to stabilize. Additionally, the lack of long-term follow-up data prevents assessment of the sustainability and stability of the intervention effect. Sixthly, there was a paucity of objective physiological indicators. Although a large amount of subjective assessment data was collected, objective physiological markers such as cortisol levels and inflammatory cytokines were not measured. These indicators may better reflect patients’ true physiological status and stress levels. The absence of these objective data limits in-depth understanding of the underlying intervention mechanisms. Lastly, despite rigorous methodological optimization and standardized statistical procedures adopted in the present study, several inherent limitations should be acknowledged. Notably, although PSM was utilized to balance baseline confounding variables and minimize selection bias, the sample size of the matched control group remained relatively small. Correspondingly, the effect sizes observed across physical, psychological, and wound healing outcomes were comparatively large in this single-center cohort. Such pronounced intervention effects may be partially attributed to residual unmeasured confounding factors, including individual differences in family socioeconomic status, informal caregiver adherence, and variations in patients’ daily self-behavioral management, which could not be fully eliminated even after PSM adjustment. Additionally, the relatively limited sample capacity may amplify the statistical effect size and overestimate the true population intervention efficacy. Therefore, the substantial beneficial effects identified in this study should be interpreted with caution. Further multicenter studies with enlarged sample sizes and stricter control of potential residual confounders are required to validate the stability and generalizability of the current findings, thereby refining the evidence basis for the clinical application of this cloud-based family engagement care model.

## 6 Conclusion

To summarize, the cloud-based family engagement care model can significantly improve the quality of life and mental health status of patients with chronic difficult-to-heal traumatic tumours. Future studies should conduct multicenter prospective randomized controlled trials to further validate the conclusions of this study.

## Data Availability

The original contributions presented in the study are included in the article/supplementary material, further inquiries can be directed to the corresponding author/s.
